# Minimally Invasive Versus Open Pancreatoduodenectomy: A Systematic Review and Meta-Analysis of Randomized Controlled Trials

**DOI:** 10.1097/AS9.0000000000000656

**Published:** 2026-03-25

**Authors:** Johannes M. A. Toti, Raffaello Roesel, Adrian T. Billeter, Beat P. Müller, Philip C. Müller, Christoph Kuemmerli

**Affiliations:** From the * Department of Surgery, Clarunis – University Centre for Gastrointestinal and Hepatopancreatobiliary Diseases, Basel, Switzerland; † Department of Surgery, Regional Hospital of Bellinzona e Valli, Bellinzona, Switzerland.

**Keywords:** laparoscopic, minimally invasive surgery, pancreas, pancreatoduodenectomy, robotic

## Abstract

**Objective:**

To compare perioperative outcomes of minimally invasive pancreatoduodenectomy (MIPD) to open pancreatoduodenectomy (OPD) using evidence from randomized controlled trials (RCTs).

**Background:**

The wider adoption of MIPD has largely been fueled by observational studies rather than high-level evidence.

**Methods:**

We searched Cochrane Central Register of Controlled Trials, MEDLINE, and Web of Science for RCTs comparing MIPD with OPD in adult patients with benign or malignant conditions requiring elective pancreatoduodenectomy. The primary outcomes were 90-day mortality, the comprehensive complication index, Clavien-Dindo grade ≥III complications, and hospital length of stay (LOS). Secondary outcomes included postoperative pancreatic fistula (POPF), delayed gastric emptying (DGE), postpancreatectomy hemorrhage (PPH), blood loss, reoperation, operative time, and oncologic outcomes. Data were pooled as odds ratios or mean differences using a random-effects model. Risk of bias was assessed using the Cochrane risk of bias tool, and the certainty of evidence was evaluated according to the Grading of Recommendations Assessment, Development and Evaluation approach (PROSPERO ID: CRD42024592919).

**Results:**

Ten RCTs with a total of 1794 patients were included. Meta-analysis showed there were no significant differences regarding 90-day mortality, Clavien-Dindo ≥3 complications, POPF, DGE, PPH, reoperation, readmission, or oncologic outcomes between MIPD and OPD. LOS was reduced for MIPD. No clinically relevant differences were found in the subgroup analyses of laparoscopic and robotic pancreatoduodenectomy. Certainty of evidence was moderate to low.

**Conclusions:**

MIPD showed no clinically relevant advantages over OPD. These findings were consistent both for the robotic and laparoscopic approach.

## INTRODUCTION

The benefits of minimally invasive surgery in abdominal procedures are well established, including reduced abdominal wall trauma, decreased postoperative pain, and faster return to normal activities. These advantages have been supported by level I evidence for a variety of operations, and minimally invasive approaches have consequently become the standard of care for left-sided pancreatic resections.^1–4^ In contrast, the role of minimally invasive techniques in more complex procedures remains less clearly defined, as postoperative recovery in these settings is driven primarily by organ-specific surgical morbidity and physiological stress rather than by the extent of the access trauma alone.

In this context, the indications for minimally invasive approaches are expanding with the intention of encompassing increasingly complex procedures, including pancreatoduodenectomy.^5^ Robotic pancreatoduodenectomy (RPD) was first reported in 2003,^6^ approximately a decade after the introduction of laparoscopic pancreatoduodenectomy (LPD).^7^ Both techniques offer the theoretical advantages of minimally invasive surgery, and the adoption of robotic platforms in particular has increased substantially over the past decade.^8^ Randomized controlled trials (RCTs) comparing LPD with open pancreatoduodenectomy (OPD) have demonstrated comparable perioperative outcomes and oncologic adequacy.^9^ However, robotic technology not only confers the general benefits of minimally invasive surgery but also addresses several intrinsic limitations of laparoscopy by providing enhanced dexterity, greater precision, and superior 3-dimensional visualization. Several institutions have reported the safety and feasibility of RPD in nonrandomized studies,^10–14^ although these series are inherently subject to selection bias.

Therefore, this systematic review and meta-analysis focused on RCTs comparing minimally invasive pancreatoduodenectomy (MIPD) with OPD, specifically evaluating perioperative outcomes, including 90-day mortality, major complications, postoperative LOS, and pancreas-specific morbidity.

## METHODS

### Systematic Literature Search Methodology

This review complies with the recommendations of the Cochrane handbook for systematic reviews and interventions, and specific recommendations for surgical systematic reviews, and is reported in line with the Preferred Reporting Items for Systematic reviews and Meta-Analyses guidelines.^15^ A protocol was developed a priori and was registered in an international systematic review registry (PROSPERO CRD42024592919).

### Eligibility Criteria

Only studies that fulfilled the following Population, Intervention, Comparison, Outcome, Study design criteria were included in the systematic review:

1. Population: adult patients (≥18 years) with a benign or malignant disease requiring elective pancreatoduodenectomy.
2. Intervention: MIPD.
3. Comparator: OPD.
4. Outcomes: intraoperative and postoperative outcomes.
5. Study design: RCTs.

### Systematic Literature Search

The following 3 databases were searched according to Goossen et al^16^:

1. Cochrane Central Register of Controlled Trials.
2. Medline (via Pubmed).
3. Web of Science.

The last database search was performed on November 26, 2025. In addition to the databases listed above, we conducted a search for ongoing trials (Clinical-Trials.gov, International Clinical Trials Registry Platform) and a web search for further trials. The last web search and trial database search was performed on November 11, 2025. Study authors of ongoing trials were contacted for information to gather missing information. The complete search string is shown in Supplemental Table 1, https://links.lww.com/AOSO/A579.

### Study Selection

Title and abstract screening were independently performed by 2 reviewers (J.M.A.T. and C.K.) using the predetermined eligibility criteria. After the first stage, both reviewers evaluated full texts and decided if the inclusion criteria and outcomes were met. The reference lists of the included studies were searched for further relevant studies by both reviewers. Any disagreement regarding inclusion was resolved by discussion with a third member of the review team (P.C.M.).

### Data Collection Process

The data were extracted using a predefined piloted extraction sheet. This process was performed independently by both reviewers (J.M.A.T. and C.K.). The data obtained were then compared by the reviewers, inconsistencies were discussed, and if necessary, a third party (P.C.M.) was consulted to reach a consensus.

### Data Items

Data was sought for general information (year of publication, country, and number of institutions); study participant characteristics (age, disease, and American Society of Anesthesiologists status), and inclusion and also exclusion criteria; type of intervention (laparoscopic, robotic, or open approach); and surgeon experience in both techniques used.

Severe complications were defined as Clavien-Dindo grade ≥3.^17^ Length of hospital stay (LOS) was considered from the day of surgery to discharge, excluding readmissions unless otherwise specified. R0 resection was defined as a tumor-free margin ≥1 mm, and lymph node yield was expressed as the total number of nodes retrieved. Mortality was reported as 90-day mortality.

### Risk of Bias in Individual Studies

Risk of bias in the individual studies was assessed independently by 2 reviewers (J.M.A.T. and C.K.) using the Cochrane Collaboration tool for assessing risk of bias. Other than the 6 domains defined by the risk of bias tool, surgeon experience for the performed operation, and sources of funding^18^ were added as potential threats to validity. Disagreements regarding the risk of bias assessment were resolved with a third party (P.C.M.). As blinding has been shown to be difficult in surgical studies and the impact of a lack of blinding is not yet clear,^19^ we assessed multiple domains (blinding of study participants, standardization of treatment, characteristics of outcomes) to reach an adequate bias assumption. Funnel plots Eggers test regression were used to assess publication bias, being aware that due to the low number of studies, there is a risk of a type II error of not detecting a publication bias when in fact there is one.^15^

### Certainty in Evidence

Certainty in evidence was assessed using the Grading of Recommendations Assessment, Development and Evaluation approach.^20^This was done independently by 2 reviewers (J.M.A.T. and C.K.) using the Grading of Recommendations Assessment, Development and Evaluation Pro Software (McMaster University and Evidence Prime Inc, Ontario, Canada). If the primary reviewers were not able to reach a consensus, a third party (P.C.M.) was included.

### Statistical Analysis

All analyses were performed using R version 4.2.3, R Core Team (2023) (R: A language and environment for statistical computing. R Foundation for Statistical Computing, Vienna, Austria. URL https://www.R-project.org/) with the “meta” package.^21^ Pooled analyses were carried out when all studies reported the outcome stratified by RPD and LPD as well as overall. The number of events, group size, and central tendency with dispersion were used to calculate point estimates. Estimates of sample means and standard deviation were calculated using the “meta” package if only median, range, or interquartile range were available, and effect sizes were converted when only precalculated data were reported.

The Sidik-Jonkman method was used to estimate the between-study heterogeneity for binary and the restricted maximum likelihood for continuous effect estimates. We then calculated pooled effect sizes by the inverse variance method. The threshold for statistical significance was set at *P* = 0.05. The *I*^2^ statistic was used to measure heterogeneity among trials in each analysis. *I*^2^ was considered representative of the severity of heterogeneity. The random-effects model to pool effect size was a priori chosen due to the assumed between-study heterogeneity. A sensitivity analysis was conducted by a leave-one-out approach.

Trial sequential analyses (TSA) were conducted. TSA uses the cumulated sample size of included trials and the pooled effect size to control the risks for type I and II error and provide an estimate of the required sample size. The most relevant pancreatectomy-specific outcome, postoperative pancreatic fistula (POPF), was selected. Information size calculations were performed to calculate the required sample size and the number of additional participants required to detect a significant difference. The TSA was conducted with an alpha level of 5% and a beta of 80%, and heterogeneity adjusted by the observed I^2^ statistics. The O’Brien—Fleming alpha-spending function was used. The alpha-spending boundaries were based on the pooled effect sizes from the meta-analysis using random effects.

## RESULTS

### Study Selection

**FIGURE 1.**
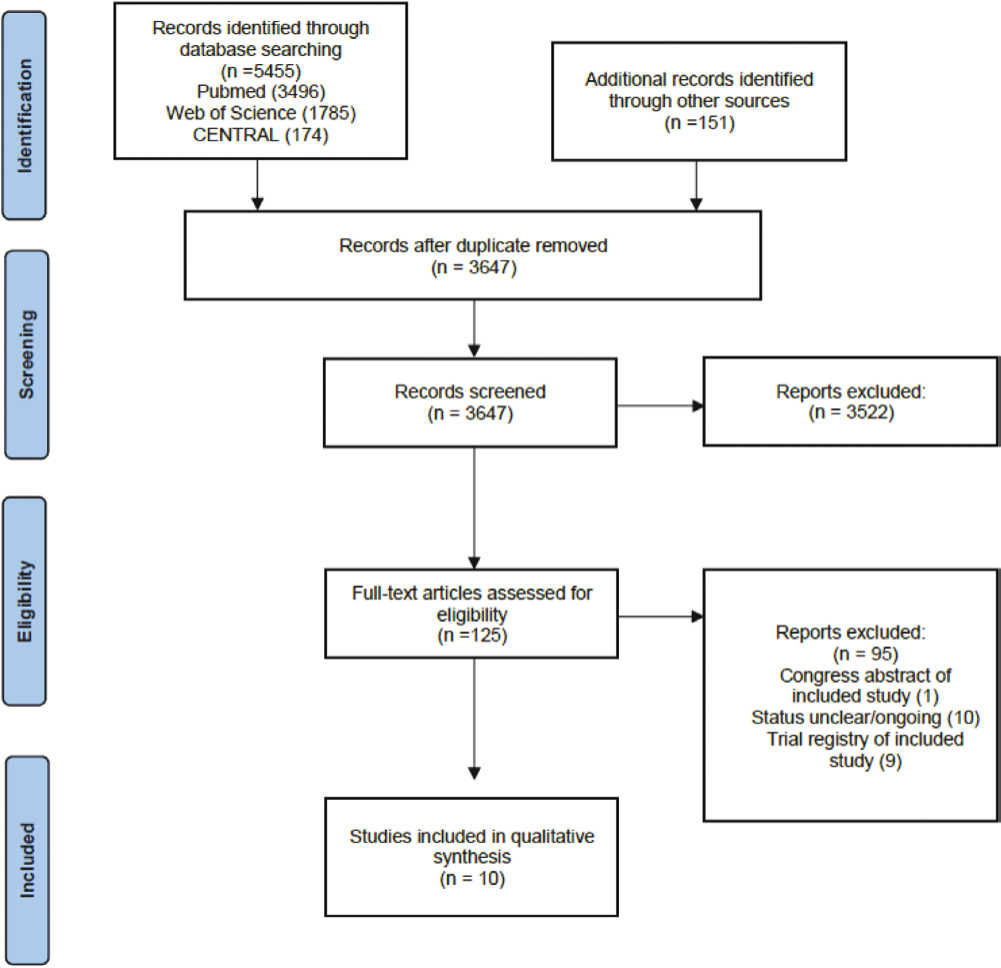
PRISMA flow diagram. PRISMA indicates Preferred Reporting Items for Systematic reviews and Meta-Analyses.

In all 3647 studies were title and abstract-screened for inclusion. Of these, 3522 were excluded due to ineligibility. One hundred twenty-five studies were full-text screened. Of these, 95 were excluded. Of the remaining studies, 10 were excluded as they presented the trial registry of an included study, one was a congress abstract of an included study, and 10 studies were ongoing (CTRI/2013/09/004016, NCT03747588, NCT04171440, NCT03722732, ChiCTR1900028686, NCT02807701, NCT05463328, NCT06661135, ChiCTR1900024788, and NCT04400357). Finally, 10 studies were included.^22–31^ The full search process is presented in Figure 1.

### Study Characteristics

The 10 trials, including 1794 patients (range 15–594), were conducted across 2 continents (Asia and Europe). Two hundred eighty patients had RPD, 662 LPD, and 852 OPD. Of the included studies, 2 compared RPD with OPD, 1 MIPD with OPD, and 7 LPD with OPD. Four were open-label monocentric, and 6 were multicentric studies. All studies included adult patients suitable for elective pancreatoduodenectomy (both MIPD and OPD) for any indication. Inclusion and exclusion criteria were similar for the studies except for the DIPLOMA 2 trial, which excluded patients with body mass index > 35 kg/m^2^ (Supplemental Table 2, https://links.lww.com/AOSO/A580). Among the included trials, only 2 studies^22,29^ reported cost analyses, both comparing RPD and OPD. Costs were reported in detail as procedure-related costs and overall in-hospital costs, explicitly excluding the acquisition cost of the robotic system. In the EUROPA trial, the median total hospital cost was €20,421 (IQR 18,203–23,605) for RPD and €16,275 (IQR 14,551–18,992) for OPD (*P* < 0.001). Similarly, overall in-hospital costs were higher in the RPD group (€33,502 ± 22,314) compared with OPD (€21,429 ± 12,427), corresponding to a mean difference (MD) of €12,073 [95% confidence interval (CI): 2932–21,213; *P* = 0.011]. In the Liu et al trial, all values were converted into Euros (1 EUR = 7.8 CNY). Median total hospital costs were €19,400 for RPD and €16,240 for OPD, resulting in a median difference of €3170 (95% CI: €1720–€4685). Median hospital stay-related costs were €14,020 for RPD and €16,240 for OPD, with a nonsignificant median difference of €2140 (95% CI: −€3595–€593). No LPD trials included cost data. Details on study characteristics are summarized in Table 1. Patient and operative characteristics as well as histological findings are presented in Table 2.

**TABLE 1.** Study Characteristics

RPD Versus OPD				LPD Versus OPD						
Study Information	EUROPA	Liu Trial	DIPLOMA 2	PLOT	PADULAP	LEOPARD-2	Bhingare Trial	Wang-1 Trial	Wang-2 Trial	Yoon Trial
First author	Klotz R	Liu Q	De Graaf N	Palanivelu C	Poves I	van Hilst J	Bhingare P	Wang M	Wang M	Yoon Y
Year	2024	2024	2025	2017	2018	2019	2019	2021/2024	2023	2024
Region	Germany	China	Europe	India	Spain	Netherlands	India	China	China	China
Design	Open-label, monocentric	Multicentric	Multicentric, patient-blinded	Open-label, monocentric	Open-label, monocentric	Multicentric patient-blinded	Open-label, monocentric	Open-label, multicentric	Open-label multicentric	Open-label multicentric
Primary outcome	CCI	LOS	CCI	LOS	LOS	Functional recovery	LOS	Short-term outcomes	Short-term outcomes	Functional recovery
Patients randomized	81	164	302	64	66	105	30	656	200	252
Patients analyzed	62	161	288	64	61	99	30	594	200	235
Conversion rate	21%	4%	8%	3%	24%	20%	NA	4%	2%	NA
Method of analyzing	mITT, PP	mITT	mITT, PP	ITT	mITT	mITT	ITT	mITT, PP	mITT, PP	mITT, PP
Number of surgeons	2 RPD 13 OPD	5	NA	2	1 LPD 2 OPD	9	2	14	NA	NA
Experience MIPD	≥40	>40	≥60	≥25	≥15	≥20	ND	≥104	≥104 LPD	≥20
Experience OPD	≥40	>60	≥60	≥100	NA	>50	NA	≥104	NA	≥100
Funding	Nonindustry funded	Nonindustry funded	Industry and nonindustry funded	Nonindustry funded	Nonindustry funded	Nonindustry funded	Nonindustry funded	Nonindustry funded	Nonindustry funded	Nonindustry funded

CCI indicates comprehensive complication index; ITT, intention to treat; LOS, length of stay; LPD, laparoscopic pancreatoduodenectomy; mITT, modified ITT; OPD, open pancreatoduodenectomy; PP, per protocol; RPD, robotic pancreatoduodenectomy.

**TABLE 2.** Study Population Characteristics, Operative Characteristics, and Histological Findings

Patient Characteristics	EUROPA Trial		Chinese Trial		DIPLOMA 2 Trial		PLOT Trial		PADULAP Trial		LEOPARD-2 Trial		Bhingare Trail		Wang-Quin Trial		Min-Wang Trial		Yoon Trial	
	RPD	OPD	RPD	OPD	MIPD	OPD	LPD	OPD	LPD	OPD	LPD	OPD	LPD	OPD	LPD	OPD	LPD	OPD	LPD	OPD
Age, y	64.7†	62.6†	62†	60†	70†	68†	57.8*	58.6*	69*	70*	67†	66†	50–59‡	60–69‡	61†	60†	61.9*	60.7*	62.5*	63.2*
Men (%)	59	49	57	60	59	57	56	69	41	69	40	51	1:0.30§	1:0.30	58	65	61	61	57	57
BMI (mean)	27	27	23	23	25†	25†	25	22	24	26	25	26	26	23	22	22	23	22	24	24
ASA 3 status (%)	38	39	7	10	34	33	6	9	41	52	26	33	0	20	23	19	27	24	12	14
Operative characteristics																				
Vascular resection (%)	17	15	4	5	3	5	3	9	13	14	10	4	0	0	3	3	2	4	NA	NA
Soft pancreas (%)	45	36	53	47	33	31	NA	NA	66	41	66	39	ND	ND	16	58	44	39	NA	NA
Histological findings																				
Tumor size (mm) (%)	62	53	63	59	25†	25†	33*	36*	24†	29†	26*	26*	33	36	24*	25*	29*	29*	NA	NA
Ampullary carcinoma (%)	6	11	15	10	21	18	47	34	16	7	24	12	27	33	8	8	0	0	10	14
Pancreatic cancer (%)	75	56	33	38	31	27	9	25	47	72	28	31	53	47	31	37	97	98	27	19
Biliary carcinoma (%)	6	11	12	20	16	14	13	19	13	7	10	16	13	7	18	16	0	0	19	17
Duodenal carcinoma (%)	0	0	7	11	6	8	31	22	0	0	6	8	7	13	20	17	0	0	3	1
IPMN (%)	0	17	12	6	8	6	0	0	3	3	16	18	0	0	2	3	0	0	22	31
Other (%)	13	7	20	15	16	20	0	0	22	10	16	14	0	0	21	19	3	2	19	18

* Mean.

† Median.

‡ Most represented interval.

§ M:F ratio of the total group.

ASA indicates American Society of Anesthesiologists; BMI, body mass index; IPMN, intraductal papillary mucinous neoplasia; NA, not assessed.

### Risk of Bias Within Studies

**FIGURE 2.**
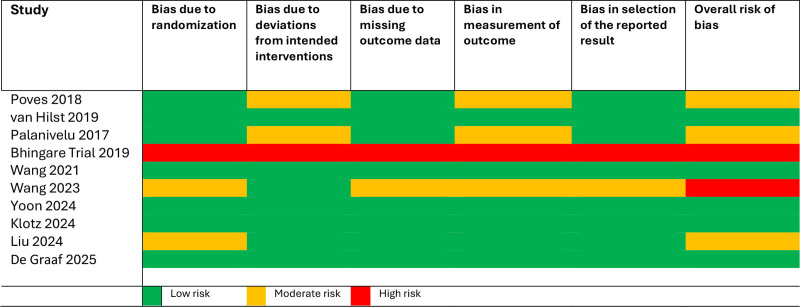
Risk of bias assessment.

All studies had a low risk of bias due to confounding, in the classification of interventions, in the selection of participants, due to deviations from intended interventions, due to missing data, in the measurement of the outcome, and selection of the reported result, except for 2 studies.^23,30^ The study by Wang et al, reported an incomplete randomization process, limited information on outcome assessment, and partial presentation of secondary endpoints resulting in an overall high risk of bias, while the study by Bhingare et al is a small single-center trial with only 30 patients, unclear allocation methods, unblinded investigators, outcomes assessed not according to established standards in the literature, and no trial registration, leading to the study ultimately classified as a high risk of bias (Supplemental Table 2, https://links.lww.com/AOSO/A580). All 10 randomized trials reported no industry-related funding or conflicts of interest, except for 2,^27,31^ which disclosed financial support from Intuitive Surgical, Inc. (Sunnyvale, CA), and the investigator-initiated LEOPARD-2 trial received funding from Ethicon Endo-Surgery (Johnson & Johnson Family of Companies, New Brunswick, NJ). Six studies^22–24,28,29,31^ reported public or academic funding, while 5^25–27,30^ declared no external financial support, indicating no detectable bias attributable to industrial involvement (Fig. 2).

As another relevant factor, we considered the experience of the surgeons involved. All trials were conducted in so-called “high-volume centers.” However, some of the earlier laparoscopic trials^26,27^ reported limited surgeon experience, which should be regarded as a potential source of bias. Only 2 trials^25,30^ did not provide information regarding the surgeon’s level of experience, which is therefore considered a relevant source of bias. In contrast, the RPD trials required a previous experience of at least 40 robotic and 60 open procedures.^22,29,31^ Funnel plots and Egger’s test indicated a low risk of publication bias (Supplemental Figure 1, https://links.lww.com/AOSO/A577).

### Main Outcomes

#### 90-Day Mortality

**FIGURE 3.**
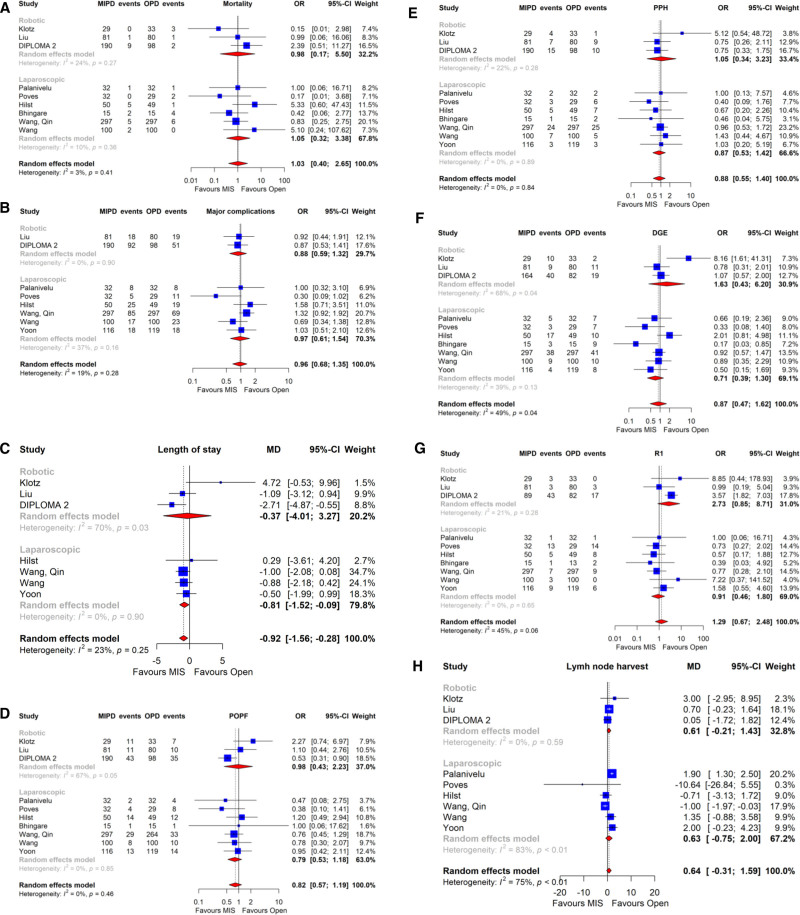
Forrest plots for main outcomes: Mortality (A), major complications (B), hospital stay (C), pancreatic fistula (D), hemorrhage (E), delayed gastric emptying (F), R0 resection (G), and lymph node harvest (H).

Ninety-day mortality was reported in all trials (1794 patients). Three trials reported overall mortality. The LEOPARD-2 trial reported complication-related mortality and cancer-related mortality separately. All observed deaths were complication related. Notably, the LEOPARD 2 trial was halted due to mortality-related safety concerns. There was no difference between MIPD group and the OPD group in postoperative mortality [odds ratio (OR) (95% CI) 1.03 (0.40–2.65); *P* = 0.952] with low heterogeneity (*I*^2^ = 3%). There was no difference comparing the subgroups of RPD [OR 0.98 (0.17–5.50)] and LDP [OR 1.05 (0.32–3.38)] with OPD (Fig. 3A). The certainty of evidence was deemed to be moderate (Supplemental Table 3, https://links.lww.com/AOSO/A581).

#### Clavien-Dindo ≥3 Complications

Clavien-Dindo ≥3 complications were reported in all except 2 trials (1702 patients). The Europa trial expressed postoperative complications without Clavien-Dindo grading. No differences were measured between MIPD and OPD [OR 0.96 (0.68–1.35); *P* = 0.794], with low heterogeneity (*I*^2^ = 19%). There was no difference comparing the subgroups of RPD [OR 0.88 (0.59–1.32)] and LDP [OR 0.97 (0.61–1.54)] with OPD (Fig. 3B). The certainty of evidence was deemed to be moderate.

#### Length of Stay

LOS was fully reported in 7 trials (1639 participants). The analysis of total MIPD cases revealed a shorter LOS for MIPD compared to OPD by a day [MD −0.92 (−1.56 to −0.28); *P* = 0.005] with a low level of heterogeneity (*I*^2^ = 23%). In the subgroup analysis, a reduced LOS by 1 day was only found for the LPD subgroup [MD −0.81 (−1.52 to −0.09)] with a moderate heterogeneity (*I*^2^ = 70%). RPD and OPD showed a comparable LOS [MD −0.37 (−4.01–3.27)] with no heterogeneity (*I*^2^ = 0%) (Fig. 3C). The certainty of evidence was deemed to be low.

### Secondary Outcomes

#### Pancreas-Specific Outcomes

##### Postoperative Pancreatic Fistula

POPF was reported in all trials (1794 patients). There was no significant difference between MIPD and OPD [OR 0.82 (0.57–1.19); *P* = 0.296] with no heterogeneity (*I*^2^ = 0%). There was no difference comparing the subgroups of RPD [OR 0.98 (0.43–2.23)] and LPD [OR 0.79 (0.53–1.18)] with OPD (Fig. 3D). The certainty of evidence was deemed to be moderate.

##### Postpancreatectomy Hemorrhage

Postpancreatectomy hemorrhage (PPH) was reported in all trials (1794 patients). Eight trials reported clinically relevant PPH (grade B/C) only, and 2 trials reported PPH grade A–C. The clinically relevant incidents were pooled and analyzed. There was no difference between MIPD and OPD [OR 0.88 (0.55–1.40); *P* = 0.5854] and a low level of heterogeneity (*I*^2^ = 0%). There was no difference comparing the subgroups of RPD [OR 1.05 (0.34–3.23)] and LPD [OR 0.87 (0.53–1.42)] with OPD (Fig. 3E). The certainty of evidence was deemed to be moderate.

##### Delayed Gastric Emptying

Delayed gastric emptying (DGE) was reported by all trials (1794 patients). Nine trials only reported clinically relevant DGE (grade B/C), and 1 trial reported all incidents of DGE. There was no significant difference between the 2 groups [OR 0.87 (0.47–1.62); *P* = 0.669], whereas heterogeneity was moderate (*I*^2^ = 46%). There was no difference comparing the subgroups of RPD [OR 1.63 (0.43–6.20)] and LPD [OR 0.71 (0.39–1.30)] with OPD (Fig. 3F). The certainty of evidence was deemed to be moderate.

### Oncologic Outcomes

#### R0 Resection

Resection status was reported by all trials (1633 patients). There was no significant difference between the 2 groups [OR 1.29 (0.67–2.48), *P* = 0.452], whereas heterogeneity was moderate (*I*^2^ = 45%). There was no difference comparing the subgroups of RPD [OR 2.73 (0.85–8.71), *P* = 0.090] and LPD [OR 0.91 (0.46–1.80), *P* = 0.776] (Fig. 3G).

#### Lymph Nodes Harvested

The number of harvested lymph nodes was reported by all trials (1794 patients). There was no significant difference between the 2 groups [MD 0.64 (−0.31–1.59), *P* = 0.185], whereas heterogeneity was high (*I*^2^ = 75%). There was no difference comparing the subgroups of RPD [MD 0.61 (−0.21–1.43)] and LPD [MD 0.63 (−0.75–2.00)] (Fig. 3H).

Apart from blood loss and duration of operation, further secondary outcomes of MIPD were not different compared to OPD (Table 3).

**TABLE 3.** Pooled Short-Term Outcomes of Patients Undergoing Pancreatoduodenectomy

Outcome	Compared to OPD	No. of Studies	I2, %	Pooled Results OR (CI)	*P*
Bile leak	RPD	3	0	1.18 (0.50–2.76)	0.698
	LPD	6	0	1.12 (0.60–2.10)	0.712
	MIPD	9	0	1.15 (0.70–1.88)	0.588
Blood loss	RPD	3	0	−97 (−127 to −67)	<0.001
	LPD	5	91	−103 (−175 to −31)	0.005
	MIPD	8	86	−99 (−142 to −55)	<0.001
Duration of operation	RPD	3	94	24 (−44–94)	0.483
	LPD	6	81	54 (32–75)	<0.001
	MIPD	9	87	47 (18–75)	0.011
Reoperation	RPD	3	0	1.09 (0.52–2.26)	0.821
	LPD	6	0	0.94 (0.38–2.30)	0.891
	MIPD	9	0	0.99 (0.54–1.81)	0.966
Readmission	RPD	3	0	1.22 (0.76–1.95)	0.418
	LPD	7	0	0.98 (0.54–1.77)	0.946
	MIPD	10	0	1.08 (0.71–1.63)	0.733
R1 resection	RPD	3	21	2.73 (0.85–8.71)	0.090
	LPD	7	0	0.91 (0.46–1.80)	0.776
	MIPD	10	45	1.29 (0.67–2.48)	0.452
LN harvest	RPD	3	0	3.6 (−0.21–1.43)	0.143
	LPD	6	83	0.63 (−0.75–2.00)	0.373
	MIPD	9	75	0.64 (−0.31–1.59)	0.185

CI indicates confidence interval; LN, lymph node; LPD, laparoscopic pancreatoduodenectomy; MIPD, minimally invasive pancreatoduodenectomy; OR, odds ratio; RPD, robotic pancreatoduodenectomy.

### Trial Sequential Analysis

The TSA showed that a total of 5168 patients would need to be randomized to detect a decrease in the odds of developing POPF of 21% when comparing MIPD to OPD. Therefore, the power of 1761 patients was insufficient, and further trials would be required (Supplemental Figure 2, https://links.lww.com/AOSO/A578).

## DISCUSSION

This study summarizes the highest available evidence comparing MIPD with OPD based on data from 10 RCTs. Clinically relevant short term surgical morbidity and oncologic endpoints did not differ between OPD and MIPD as well as between the 2 subgroups of RPD and LPD, compared to OPD (Table 4). MIPD decreased LOS by a day and was associated with less blood loss.

**TABLE 4.** Summary of Findings: Robotic Versus Open Pancreatoduodenectomy

Outcomes	Assumed Risk OPD Per 1000	Corresponding Risk RPD Per 1000 (95% CI)	Difference Per 1000	Relative Effect (95% CI)	Number of Studies	Certainty of Evidence (GRADE)
Mortality	23	24	1 (−14–36)	1.03 (0.4–2.65)	10	Moderate
Complications	271	247	−25 (−89–62)	0.96 (0.68–1.35)	8	Moderate
Length of stay	13.8	12.9	−0.9 (−1.6 to −0.3)	–	7	Low
Pancreatic fistula	164	135	−29 (−70–31)	0.82 (0.57–1.19)	10	Moderate
Postpancreatectomy hemorrhage	82	73	−10 (−35–29)	0.88 (0.55–1.4)	10	Moderate
Delayed gastric emptying	148	132	−16 (−66–65)	0.87 (0.47–1.62)	10	Moderate
R0 Margin	72	86	14 (−23–80)	1.29 (0.67–2.48)	10	Low
Lymph node harvest	16	17	1 (0–2)	–	9	Low
Operative time	338	385	47 (19–75)	–	9	Moderate
Blood loss	365	265	−99 (−143–56)	–	8	Low
Bile leak	57	65	8 (−16–46)	1.15 (0.7–1.88)	9	Moderate
Reoperation	52	52	0 (−22–38)	0.99 (0.54–1.81)	9	Low
Readmission	92	97	6 (−25–49)	1.08 (0.71–1.63)	10	Low

GRADE indicates Grading of Recommendations Assessment, Development and Evaluation.

The findings from this up-to-date meta-analysis are not unexpected when following the theorem that the access trauma is surpassed by the organ-specific morbidity in case of more complex surgical procedures, such as major pancreatic surgery. The body of evidence is complicated by the changing preference in minimally invasive approach. Most contemporary trials evaluate the robotic access,^22,29,31^ while older RCTs compared LPD with the open approach.^23–28^ Compared to RPD, LPD is technically more demanding, less ergonomic, and was adopted by a minority of very experienced laparoscopic hepatopancreatobiliary centers. Interestingly, the subgroup analysis in this study found no clinically relevant differences between RPD and LPD except for a reduced LOS for LPD.

The learning curve for MIPD has been clearly defined, reporting performance stabilization after approximately 39 LPD and 25 RPD, while mastery is generally reached after more than 100 MIPD procedures.^32^ According to these thresholds, 5 trials^22,23,28,29,31^ were conducted after the learning phase, whereas one^24^ included both surgeons during and after the learning phase, and 3^25–27^ were performed during the initial learning phase. Notably, only 1 trial reported surgeon experience exceeding 120 MIPD,^24^ approaching a mastery phase; however, the majority of available randomized trials were still executed in earlier stages of technical maturation, indicating that current evidence largely reflects evolving rather than fully consolidated expertise.

Previous meta-analyses from laparoscopic randomized evidence reported similar results without clinically relevant advantages of MIPD over the open approach, except for reduced blood loss, while raising concerns about safety.^9^ Later syntheses, including both randomized and nonrandomized studies, confirmed feasibility but consistently showed no improvement in major outcomes.^33,34^ Analyses restricted to robotic procedures, all nonrandomized, suggested shorter hospital stay, less blood loss, and lower short-term mortality, but these advantages were offset by higher reoperation rates and the inherent risk of selection bias.^35^

Unlike previous reviews on LPD that emphasized learning curve effects, our interpretation highlights that after pancreatoduodenectomy (PD), outcomes are driven predominantly by the intraabdominal morbidity of the resection and reconstruction, limiting the impact of the access route. This helps to explain why minimally invasive approaches have not translated into improvements in strong clinical endpoints, though selected patients may still benefit from earlier recovery and timelier initiation of adjuvant therapy.

Traditional endpoints such as mortality and major morbidity may no longer fully capture the advantages of MIPD. While outcomes have improved and complication rates have decreased, evaluating complications individually may underestimate the overall clinical benefit. The TSA demonstrated that there is currently insufficient power to detect a difference in a single endpoint (eg, POPF) if there is truly one. Emerging approaches like the PACE,^36^ a composite endpoint that aggregates POPF, PPH, and reintervention to improve sensitivity and reduce sample size requirements, and the textbook outcome,^37^ which defines an “ideal” composite perioperative course, suggest that composite metrics may better reflect modern surgical success. To the best of our knowledge, there are currently no registry trial protocols on this topic that incorporate these composite measures. Future RCT’s should thus incorporate these multidimensional endpoints together with functional recovery endpoints that may better reflect the potential advantages of minimally invasive approaches. Oncological endpoints, such as time to initiation and completion rate of adjuvant therapy, are particularly relevant in pancreatic cancer surgery, where delays in systemic treatment compromise long-term survival.^38^ While the available RCTs are not powered to detect a difference in oncologic outcomes, more trials are currently recruiting. The ongoing DIPLOMA 2 × 2 trial will address the question of noninferiority of RPD with regard to short-term oncologic outcomes. Its primary outcome measure is radicality, defined as R0 resection and complete removal of the tumor. This trial will continue recruitment seamlessly from the DIPLOMA 2 trial. Other RCTs in progress are the Chinese PORTAL trial (NCT04400357), the Japanese SWARS trial (jRCT: 1030250312), and the Spanish SPAIN PD trial (NCT06981273). Neither assesses oncologic outcomes as the primary endpoint. Hence, long-term outcomes remain challenging to compare. Most likely, a noninferiority design would be appropriate to assess the noninferior survival or disease-free survival after RPD compared to OPD. Alternatively, and at a later stage, an IPD meta-analysis may be the best way to address a stage-stratified analysis of survival. This pooled analysis can address the heterogeneity issue of the study samples and would be sufficiently powered by including the numerous enrolled patients from all published and recruiting trials.

Despite a systematic assessment of the risk of bias,^39^ certain confounding factors specific to surgical innovation are not fully captured by this framework.^40^ Industrial sponsorship may influence study design or reporting; however, in the present analysis, only 2 trials reported evidence of industry-related funding. In contrast, the learning curve remains an often-underestimated source of bias, as surgeon experience directly affects outcomes and may distort comparisons between minimally invasive and open approaches. Moreover, even when numerical thresholds for case volume are met, the quality of technical maturation varies across centers and individual surgeons, reflecting heterogeneity in training environments and institutional experience. These considerations align with the Idea, Development, Exploration, Assessment, Long-term study framework, which advocates phased evaluation and methodological rigor in the development and introduction of new surgical technologies.^41,42^ Nonetheless, most surgical RCT’s still fail to account for learning-curve effects, clustering, and surgeon performance, limiting both internal validity and patient safety.^43^ This is particularly relevant for complex procedures such as PD, where differences in surgeon expertise and institutional readiness can substantially affect safety, as shown by premature trial termination due to excess mortality in MIPD.^27^ Furthermore, accumulating evidence demonstrates that intraoperative technical performance strongly influences postoperative and oncologic outcomes, underscoring that the true intervention in surgical trials is not only the operative approach but also the quality with which it is executed.^44,45^ Integrating structured Idea, Development, Exploration, Assessment, Long-term study-based methodology and objective assessments of surgical performance, such as video-based or artificial intelligence-assisted evaluation, would complement conventional bias assessment and ensure that minimally invasive pancreatic surgery advances through safe, reproducible, and evidence-based innovation.^46,47^

### Limitations

This meta-analysis included a limited number of randomized studies with an overall limited sample size, which may have prevented the detection of differences. To address this, our TSA assessed illustratively the required sample size for POPF to detect a difference. The numbers indicated at least 1 large RCT to detect a difference that might not be clinically meaningful anymore. Further, most trials were underpowered and methodologically heterogeneous, reducing the robustness of pooled estimates. However, a sensitivity analysis by recalculating pooled effect estimates with leaving 1 study 1 showed no difference in outcome, by either no or only a little change in effect, and no change in the significance level.

## CONCLUSION

As of the current level I evidence from RCTs, MIPD shows little advantage over OPD, especially major morbidity and mortality were not different between the approaches. The intrinsic morbidity of pancreatic resection and the persistent impact of the learning curve remain dominant determinants of outcome, highlighting the need for future trials to focus on innovative patient-centered endpoints and take into account the MIPD learning curves to capture a potential benefit of MIPD.

## ACKNOWLEDGMENTS

C.K. did acquisition of data, statistical analysis, interpretation of data, figures, drafting the article, and gave final approval. J.M.A. and C.K. did conception and design, acquisition of data, critical revision of the article, and gave final approval. R.R., A.T.B., and B.P.M. did conception and design, interpretation of data, critical revision of the article, and gave final approval. P.C.M. did conception and design, interpretation of data, figures, drafting the article, and gave final approval. The data that support the findings of this study are available on request from the corresponding author.

## Supplementary Material
