# Supplementary material for: Minimally Invasive Versus Open Pancreatoduodenectomy: A Systematic Review and Meta-Analysis of Randomized Controlled Trials
Source: Ann Surg Open. 2026 Mar 25;7(2):e656. doi: 10.1097/AS9.0000000000000656 (PMC13290216; doi:10.1097/AS9.0000000000000656)
Supplement: Supplementary file 1 [file as9-7-e656-s001.pdf]

Supplementary Table 1: Search strategy

Search string	The search was conducted using a combination of medical subject headings (MeSH) and free text words. The search strategy for Medline (via Pubmed) was the following: (minimal* invasive OR laparosc* OR robot* OR hybrid OR telerobotic* OR Vinci* OR daVinci* OR ‘‘Robotic Surgical Procedures’’) AND ((pancreatoduodenectom* OR pancreaticoduodenectomy OR ‘‘pancreatectomy’’ OR pancreaticoduodenectom* OR PPPD OR pancreatectom* OR duodenopancreatectom* OR Kausch-Whipple OR Whipple* OR ppWhipple* OR ‘‘pancreatic head resection’’ OR ‘‘Pancreatic Neoplasms/surgery’’) OR ((pancreas* OR pancreatic* OR ‘‘Pancreas’’ OR ‘‘Pancreatic Diseases’’) AND (surger* OR surgical OR operation* OR resection*))) NOT (animals NOT humans)
