# Supplementary material for: Minimally Invasive Versus Open Pancreatoduodenectomy: A Systematic Review and Meta-Analysis of Randomized Controlled Trials
Source: Ann Surg Open. 2026 Mar 25;7(2):e656. doi: 10.1097/AS9.0000000000000656 (PMC13290216; doi:10.1097/AS9.0000000000000656)
Supplement: Supplementary file 2 [file as9-7-e656-s002.pdf]

	EUROPA Trial	Chinese Trial	DIPLOMA 2 Trial	PLOT Trial	PADULA Trial	LEOPARD 2 Trial	Bhingare Trial	Min-Wang 1	Min-Wang 2	Yoon Trial
**Inclusion criteria**	Patients suitable for both RPD and OPD	Patients suitable for both RPD and OPD<br>- Age 18–75y<br>- ECOG PS 0–1<br>- ASA ≤3<br>- Resectable tumor	Patients suitable for both MIPD and OPD with upfront resectable disease	- Age 30–70y<br>- Resectable periampullary cancer<br>- No previous chemotherapy	Patients suitable for elective PD	- Elective PD<br>- Fit to undergo procedure	- Resectable cancer	- Age 18–75y<br>- Elective PD	- Age 18–75y<br>-Histologically confirmed or clinically diagnosed PDAC	- Age 19-80y<br>- ECOG 0-2
**Exclusion criteria**	Borderline resectable or unresectable tumor<br>- Distant metastases<br>- ASA score >3<br>- Participation in trial that could interfere with the intervention of this trial<br>- Language difficulties or lack of compliance	Borderline resectable tumor<br>-Neoadjuvant therapy<br>- Distant metastases<br>- Major comorbidity<br>- Synchronous malignant tumour of other organs<br>- Pregnancy	- Not fit for surgery<br>- Not able to provide informed consent<br>- 2nd cancer requiring resection<br>- Chronic pancreatitis<br>- Vascular involvement<br>- BMI >35 kg/m2<br>- Participant in another trial that could interfere<br>- Chronic pancreatitis as indication<br>- Pregnancy	Unresectable disease at the outset or during procedure	-Distant metastases<br>- Advanced tumor requiring vascular resection<br>- Rescue surgery after NAT<br>- ECOG >2<br>- Severe chronic hepatic, renal, pulmonary, or cardiac disease<br>- Hostile abdomen for MIPD<br>- Pregnancy	Involvement of major vasculature based on computed tomography<br>- Body mass index >35 kg/m2<br>- Neoadjuvant radiotherapy	Unresectable tumors at surgery<br>- Inoperable tumors receiving palliative chemo-radiation.	Distant metastases<br>- Other pancreas resection<br>- ASA score of at least 4<br>- Synchronous malignancy in other organs or a second cancer requiring resection<br>- Pregnancy<br>- Patients who underwent or required neoadjuvant chemoradiotherapy	Distant metastases<br>- Vascular invasion and vascular resection<br>- ASA score of at least 4<br>- history of another malignant disease	Distant metastases<br>- Vascular invasion<br>- Prior chemotherapy<br>- BMI >30 kg/m2<br>- Severe psychiatric / neurological disorders<br>- Alcohol or drug addiction<br>- synchronous malignancy in other organs, second cancer requiring simultaneous resection

BMI, body mass index; LA, locally advanced; NAT, neoadjuvant therapy
