# Supplementary figures and images for: Minimally Invasive Versus Open Pancreatoduodenectomy: A Systematic Review and Meta-Analysis of Randomized Controlled Trials
Source: Ann Surg Open. 2026 Mar 25;7(2):e656. doi: 10.1097/AS9.0000000000000656 (PMC13290216; doi:10.1097/AS9.0000000000000656)

**Mortality**

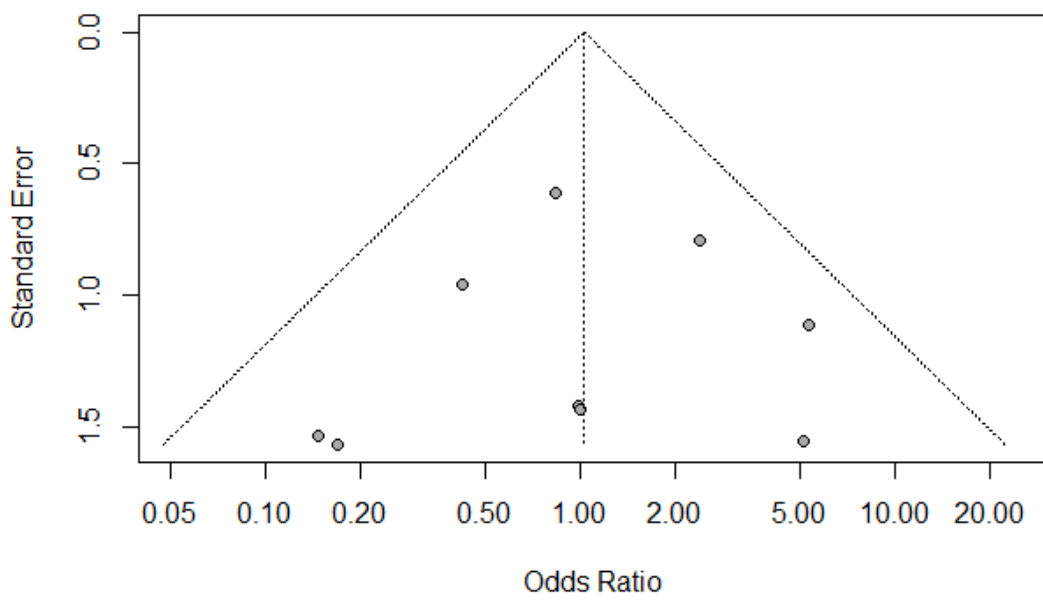

**Major complications**

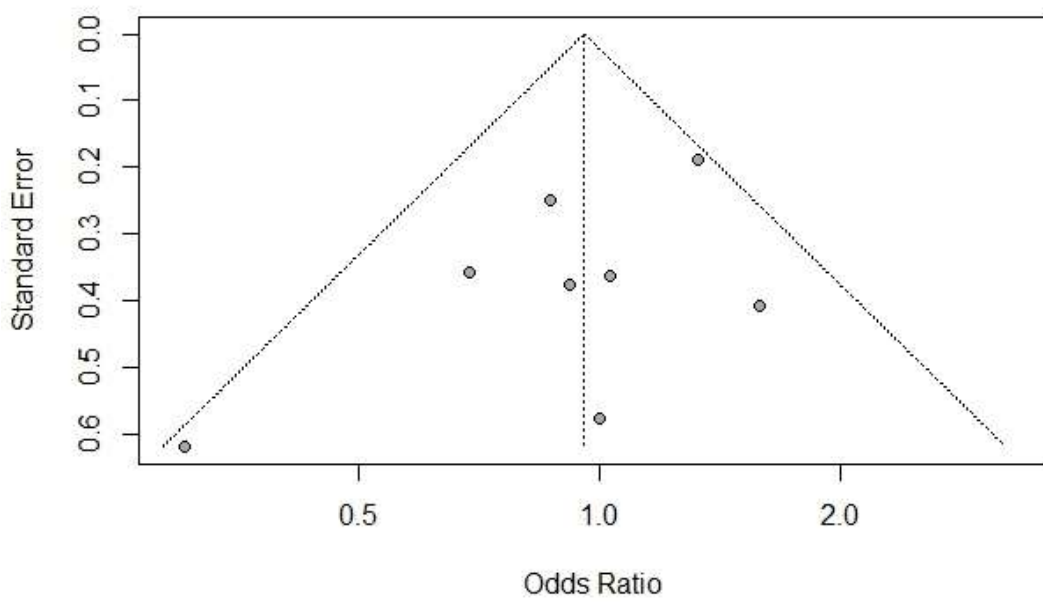

**Length of stay**

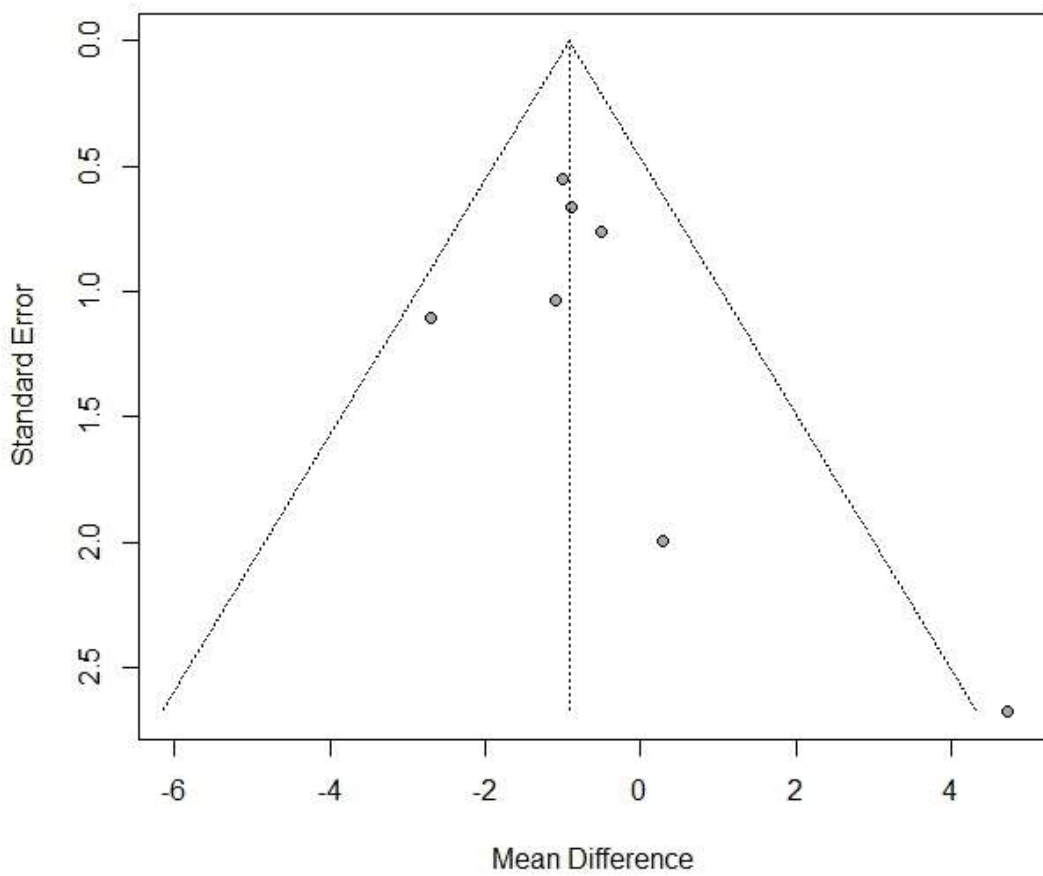

Supplement: Supplementary file 3 [file as9-7-e656-s003.pdf]
