# Supplementary material for: Minimally Invasive Versus Open Pancreatoduodenectomy: A Systematic Review and Meta-Analysis of Randomized Controlled Trials
Source: Ann Surg Open. 2026 Mar 25;7(2):e656. doi: 10.1097/AS9.0000000000000656 (PMC13290216; doi:10.1097/AS9.0000000000000656)
Supplement: Supplementary file 4 [file as9-7-e656-s004.pdf]

Supplementary Table 3: Certainty of Evidence (GRADE)

Outcome	Certainty of Evidence (GRADE)
Mortality	Moderate
Complications	Moderate
POPF (Postoperative Pancreatic Fistula)	Moderate
DGE (Delayed Gastric Emptying)	Moderate
PPH (Postpancreatectomy Hemorrhage)	Moderate
Bile leak	Moderate
Operation time	Low
Length of hospital stay	Low
Chyle leak	Very low / Not enough data
1–5 year survival	Very low / Not enough data
