# Supplementary material for: Minimally Invasive Versus Open Pancreatoduodenectomy: A Systematic Review and Meta-Analysis of Randomized Controlled Trials
Source: Ann Surg Open. 2026 Mar 25;7(2):e656. doi: 10.1097/AS9.0000000000000656 (PMC13290216; doi:10.1097/AS9.0000000000000656)
Supplement: Supplementary file 5 [file as9-7-e656-s005.pdf]

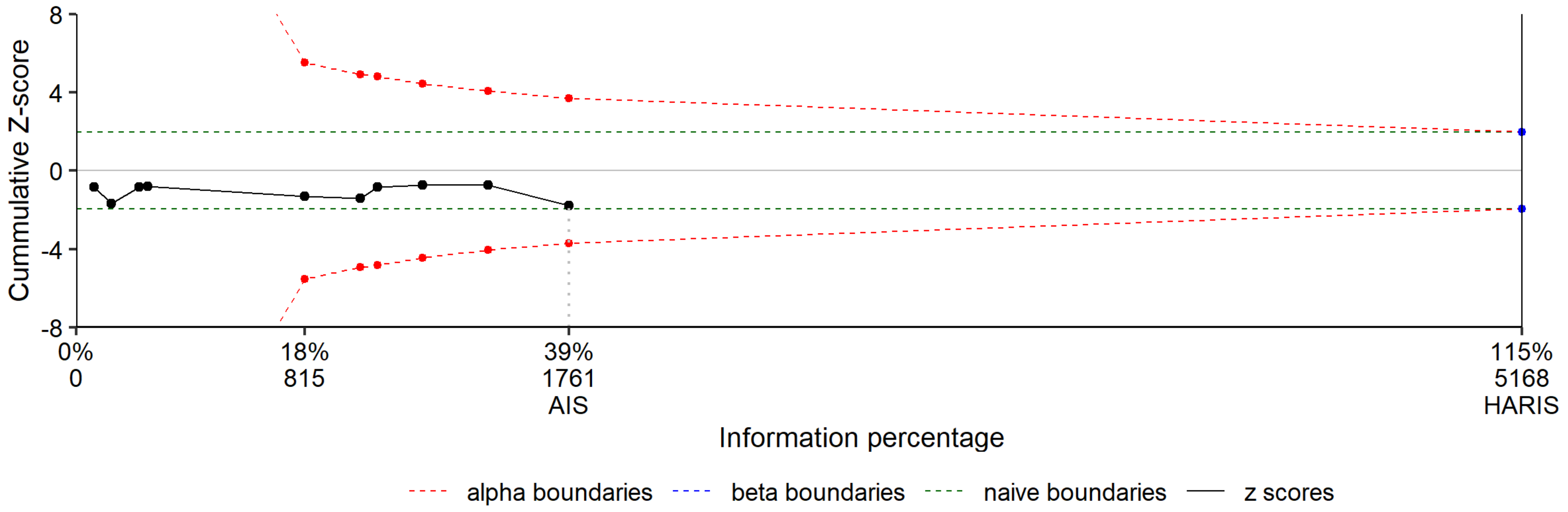

Retrospective TSA with: pc 16.4%, MVD OR 79.0%, alpha 5.0%, beta 20%. .
